# Burnout, life satisfaction, and work-related quality of life among psychologists

**DOI:** 10.3389/fpsyg.2025.1532333

**Published:** 2025-02-26

**Authors:** Silvia Morais de Santana Ferreira, Victor Zaia

**Affiliations:** Postgraduate Program in Health Sciences, Centro Universitário Faculdade de Medicina do ABC, Santo André, Brazil

**Keywords:** burnout, life satisfaction, work-related quality of life scale, psychometric validation, psychologist

## Abstract

**Introduction:**

The Work-Related Quality of Life (WRQoL) is a complex, multidimensional concept, and its assessment is challenging due to a lack of consensus on the factors involved. While the WRQoL Scale has been validated in various countries, no translation into Brazilian Portuguese existed until this study. The primary aim was to validate the Brazilian Portuguese version of the WRQoL Scale, using psychologists as the target population, and to measure burnout and life satisfaction in this group.

**Methods:**

A backtranslation process was followed, involving bilingual translators and a focus group of psychology professionals to refine the final version. A total of 610 psychologists participated, completing the Maslach Burnout Inventory, the Life Satisfaction Scale, and the WRQoL Scale via an online platform (SurveyMonkey^®^). Exploratory and confirmatory factor analyses were conducted to test and refine the factor structure of the WRQoL Scale. Spearman's correlations and group comparison analyses were also performed.

**Results:**

Seventy-six percentage of participants reported adequate life satisfaction, while 60.2% exhibited burnout symptoms, particularly in emotional exhaustion and depersonalization. Protective factors for the mental health of psychologists were identified. The final version of the WRQoL Scale included 23 items across three factors, with good internal consistency (Cronbach's alpha = 0.89 ICC = 0.96).

**Discussion:**

The WRQoL Scale - Brazilian version, demonstrated adequate psychometric properties, as evidenced by its construct validity and internal consistency. This provides evidence that the WRQoL Scale is a adequate tool that can measure work-related quality of life in psychologists.

## 1 Introduction

Occupational activity is an essential aspect of life, and when perceived as a source of satisfaction, it contributes positively to overall life satisfaction (Uchmanowicz et al., 2019). Life satisfaction is a cognitive construct that reflects how individuals evaluate their satisfaction in various life domains, such as work, leisure, relationships, health, and finances (Diener et al., 1985). The profession of psychology, in turn, demands intense emotional effort to address the inherent challenges of the role, given the nature of the professional experiences to which psychologists are exposed (Hammond et al., 2017; Råbu et al., 2016; Rodriguez and Carlotto, 2017). While psychologists often derive satisfaction from their work and are driven by a strong desire to help others, this same dedication can lead them to overlook their personal limitations, ultimately compromising their mental health (Rodriguez and Carlotto, 2017).

This emotional effort, compounded by the necessity of employing empathy without adequate training and the high expectations placed on psychologists by clients and others regarding the resolution of their issues, may contribute to the development of burnout (Villacieros et al., 2017; Weir, 2016). A systematic review (Simionato and Simpson, 2018) confirmed that psychotherapists exhibit moderate to high levels of burnout on assessments.

Burnout arises as a consequence of prolonged exposure to chronic work-related stress (Moss et al., 2016). It is characterized by three dimensions: emotional exhaustion (e.g., feelings of being overwhelmed), depersonalization (e.g., an indifferent or detached response), and reduced personal accomplishment or inefficacy (e.g., feelings of incompetence; Maslach et al., 2001). Predictors of burnout include workload, shift work, conflicting work demands, lack of control and autonomy in the work environment, insufficient social support (Casida et al., 2019; Maslach et al., 2001; Moss et al., 2016), personality traits, stress coping, beliefs, personal attitudes, and work-life imbalance (Hammond et al., 2017; Maslach et al., 2001; Moss et al., 2016; Rodriguez and Carlotto, 2017; Simionato and Simpson, 2018; Simpson et al., 2019). Professionals experiencing high levels of burnout often show impairments in their ability to provide services to patients and face personal, familial, and social challenges (Simpson et al., 2019).

High levels of life satisfaction have been associated with lower burnout rates (Bartosiewicz et al., 2020; Uchmanowicz et al., 2019), and better work-related quality of life (Sansó et al., 2020). A protective factor against burnout is adequate Work-Related Quality of Life (WRQoL), which not only inversely correlates with burnout but also encompasses a wide range of work-related experiences (Cetrano et al., 2017; Wang et al., 2019). However, the multidimensional nature of WRQoL makes achieving conceptual consensus challenging, as researchers employ varying sets of variables to measure it (Geoffrion et al., 2019; Wang et al., 2019). Commonly identified factors in WRQoL include rewards, benefits, remuneration, career prospects, workload, environmental support, work stress, and work-life balance (Akter et al., 2018; Geoffrion et al., 2019; Nowrouzi et al., 2016).

The Work-Related Quality of Life Scale (WRQoL) was developed to assess the interplay between professional and personal life domains. Initially validated among healthcare professionals, the original version comprises a 23-item, six-factor measurement model. These factors include Job and Career Satisfaction (i.e., satisfaction with the career opportunities), General Well-Being (i.e., perception that things are working well for oneself), Home-Work Interface (i.e., adequate adjustment between work and personal life), Stress at Work (i.e., feeling of pressure at work), Control at Work (i.e., participation in decisions that affect me), and Working Conditions (i.e., satisfaction with working conditions, including physical conditions), with a Cronbach's α of 0.91 (Van Laar et al., 2007).

The WRQoL scale has been translated and adapted into several languages (e.g., English, Thai, Italian, Farsi…) and used across various populations (e.g., healthcare employees, nurses, physicians…), consistently proving to be reliable (Dai et al., 2016; Gomes et al., 2013; Shabaninejad, 2012; Shukla et al., 2017; Van Laar et al., 2007). However, a Brazilian Portuguese version has not yet been developed, despite it being the sixth most spoken native language worldwide (221 million native speakers) and the ninth most spoken language overall (Paolillo and Das, 2006). Additionally, we did not find a version specifically tailored for psychologists in the existing literature—Supplementary Table 1. This study thus aimed to measure and analyze burnout (MBI-HSS), life satisfaction (EVS), and work-related quality of life (WRQoL) among psychologists and to perform the semantic and psychometric validation of the WRQoL Scale for Brazil.

Understanding the interplay between burnout, life satisfaction, and work-related quality of life is particularly important for psychologists. While they often derive satisfaction from their dedication to patient care, this same commitment can lead to emotional exhaustion, a core aspect of burnout (Lee and Akhtar, 2011).

## 2 Materials and methods

This observational, cross-sectional study followed STROBE guidelines (von Elm et al., 2007), and utilizing online data collection. It adhered to best practice guidelines for survey-based studies (Kelley, 2003), the CHERRIES protocol (Eysenbach, 2012) for reporting internet-based questionnaires. The study was divided into two main phases: (i) translating the WRQoL Scale, performing semantic, cultural and psychometric validation; (ii) and the measurement analyses of the WRQoL Scale, the MBI-HSS, and the EVS.

### 2.1 Participants

The minimum effective sample size was set at 560 participants, taking into account the assumptions of factorability and the potential to conduct confirmatory and exploratory factor analyses with different subsamples (Kliner, 2005). The data were collected between March and August 2020 via an online platform. Participants were recruited and invited through online platforms, including social media, instant messaging applications, and email.

A total of 803 psychologists agreed to participate in the survey. Of them, 193 dropped out during the completion of the instruments (86 dropped out when completing the sociodemographic questionnaire; 46, the EVS; 36, the MBI-HSS; and 35, the WRQoL), representing a sample loss of 24.03%. The final sample comprised 610 psychologists from all regions of Brazil. The inclusion criteria were as follows: (a) being a psychologist, (b) working in the field of psychology, and (c) having at least 1 year of training as a psychologist. No exclusion criteria were applied.

### 2.2 Translation and semantic validation

Prior to initiating the study, the WRQoL scale underwent translation and semantic validation. The English version was translated using a back-translation method (Beaton et al., 2000; Sousa and Rojjanasrirat, 2011). Two independent health professionals, fluent in English and native speakers of Portuguese, conducted the initial translation. Both were familiar with the study objectives, psychometric properties, and conceptual framework of the instrument. The first independent translation (version 1) was produced and subsequently refined through consensus.

This consensus version (version 2) was then backtranslated into English by a native speaker who was unaware of the study objectives and the original instrument. Three bilingual researchers compared this backtranslation to the original English version, identifying and resolving any discrepancies until both versions were indistinguishable.

Next, version 2 was translated into Brazilian Portuguese by two additional independent health professionals, fluent in English and native speakers of Portuguese (version 3). This version was compared with version 1, and both were found to be nearly identical. Consequently, version 3 was presented to a focus group of 11 participants for semantic validation. The group consisted of psychologists working in various fields, including clinical practice, education, school psychology, psychological assessment, organizational psychology, and social psychology. The objective was to check the semantic equivalence, converging meaning between sentences in the original and translated texts, idiomatic equivalence rendering idiomatic expressions as natural as possible in the target language, cultural equivalence to meet cultural aspects in the original text and adaptation to the target context, and conceptual equivalence and corresponding concepts and ideas, resulting in a final version (version 3).

Participants of the focal group were invited by the authors and included psychologists and professors at different Brazilian universities, specialists in psychometrics. Their ages ranged from 27 to 59 years-old, in a full session for 2.5 h. The final version was then the one used in the validation phase, submitted to the participants.

### 2.3 Instruments

#### 2.3.1 Sociodemographic and labor questionnaire

It included questions about age, sex, level of education, marital status, place of residence, number of children, length of experience, area of work, job satisfaction (e.g., how satisfied are you with your profession?), and self-care (e.g., do you engage in any leisure activities?).

#### 2.3.2 Work-related quality of life (WRQoL) scale

The translated and adapted version that was administered to the participants comprises 23 items. The responses are measured on a Five-point Likert-type scale (e.g., 1 = *strongly disagree*, 5 = *strongly agree*). The higher the score, the higher the work-related quality of life (Van Laar et al., 2007). The score was calculated based on the psychometrically validated version in the present study; therefore, we consider the present final version, which is structured across three factors: Work-related Wellbeing, Job-career Satisfaction, and Work Environment. The final version is available in Supplementary Table 2.

#### 2.3.3 Life satisfaction scale (ESV)

It assesses the judgments people make about their overall life satisfaction (Gouveia et al., 2005). It comprises fie items and responses are measured on a Seven-point Likert-type scale, ranging from 1 (*strongly disagree*) to 7 (*strongly agree*). The possible score range is from 5 (low satisfaction) to 35 (high satisfaction), with a neutral point of 20 (Diener et al., 1985).

#### 2.3.4 Maslach burnout inventory (MBI-HSS)

It assesses professional burnout (Cardoso et al., 2018). It comprises 22 items divided into three domains: Emotional Exhaustion (EE), Depersonalization (DP), and Professional Accomplishment (PA). The answers are provided on a Five-point Likert-type scale ranging from 1 (*never*) to 5 (*always*). For the Emotional Exhaustion and Depersonalization subscales, higher scores indicate greater degree of burnout. However, lower scores on the Professional Accomplishment subscale indicate higher degree of burnout. Scale scores are considered separately (Maslach et al., 1996).

### 2.4 Procedures

The instruments were entered into SurveyMonkey^®^, which was used as a tool for online data collection. The initial invitation to participate in the study was made through social media to psychology professionals, regional professional councils, and psychologist-related organizations. The sample was characterized as non-probabilistic, specifically using a snowball sampling technique. Participants had access to the consent form, and only after accepting it, could they access the scale. Participants were also asked to invite at least one more colleague, if possible, to participate in the study.

### 2.5 Ethical aspects

Participation was voluntary and anonymous. All participants provided informed consent, and the study was conducted in accordance with the Declaration of Helsinki. The study was also approved by the Research Ethics Committee of *Centro Universitário de Juazeiro do Norte* under national registration number 1.878.066.

### 2.6 Data analysis

The data analysis was performed using R 4.4.1. The sample was characterized by descriptive statistics (frequency, mean, median, mode, minimum and maximum values, and standard deviations), differentiating categorical variables from continuous variables. We assessed the data for univariate (Kolmogorov-Smirnov) and multivariate (Henze-Zirkler's Test and Mardia's Test) normality. Missing values were examined and replaced with the median, applying this procedure only when the percentage of missing responses did not exceed 30% for each measure (6 missing values were identified). If the missing values exceeded 30% in at least one measure, the participant would be excluded from the study, no participant was excluded for this reason.

Exploratory Factor Analysis (EFA) and Confirmatory Factor Analysis (CFA) were used. The database was randomly divided using an R command, selecting 280 participants for EFA (subsample 1) and the remaining 330 participants CFA (subsample 2). The subsamples were tested for homogeneity using chi-square and Mann-Whitney tests, which indicated homogeneity across all descriptive variables, except for income, where higher levels were more prevalent in the CFA subsample.

To perform the EFA, we considered subsample 1 and all 24 items of the WRQoL scale. The Kaiser-Meyer-Olkin test (KMO) (>0.50) and Bartlett's test were used to check if the correlation matrix is not an identity matrix as both tests indicate if a factor analysis is allowed (Hair et al., 2009; Peres-Neto et al., 2005). The number of factors to retain was tested with the Horn's Parallel Analysis (Chen and Weng, 2023; Watkins, 2000). The pattern matrix was checked and the criteria for item inclusion was the primary factor load > |0.30| (Horn, 1965; Mair, 2018). For a factor to be considered viable, it had to contain at least three items (Tabachnick et al., 2019).

Reliability was assessed for internal consistency with Cronbach's alpha (cut ≥ 0.60), Interclass composite coefficient (≥0.80) and Omega (ω) (≥0.70) (Kline, 2015; Perreira et al., 2018; Streiner et al., 2016; Tabachnick et al., 2019).

In subsample 2, CFA was conducted to confirm the structure identified in the EFA. The model was estimated using the diagonally weighted least squares (DWLS). The fitted model was evaluated with the significance level of *p* < 0.05, the root-mean-square approximation error (RMSEA) (< 0.08), the comparative fit index (CFI) and Tucker-Lewis' index (TLI) (>0.95), and χ^2^/df (< 5.0) (Hu and Bentler, 1999). In cases where the model required adjustments, modification indices were examined, revealing potential error covariances. In such cases, these covariances were incorporated into the model by considering the highest modification indices until an adequate model fit was achieved (Brown and Moore, 2012).

Given the non-normal distribution of the data, Spearman's Rho was conducted to test the potential correlation of the WRQoL scale and the other instruments. Criterion validity was assessed by considering the other measures used in the study, namely the measures of life satisfaction and burnout. For comparing sociodemographic characteristics, group comparisons were performed using the following tests, Chi-square, the Mann-Whitney *U* test was utilized for variables with two categories, while the Kruskal-Wallis test was employed for variables with three categories (Hoermann et al., 2020). A statistical significance level of *p* ≤ 0.05 was used as a parameter to interpret the results. The correlation coefficient of 0.10 was considered small, 0.30, medium, and 0.50, large. Additionally, Cohen's *d* of 0.20 was considered small, 0.50, medium, and 0.80, large in effect size (Cohen et al., 1988).

## 3 Results

### 3.1 Sample characterization

Among the participants, 87% are women, 55.6% are married, and 55.2% have a low income. The majority of professionals hold a specialization (68.9%), 46.7% are self-employed, and 56.1% hold more than one job. On average, participants have been working in their respective fields for 12.2 (±9.4) years and report a weekly workload of 31.9 (±13.8) hours. With regard to health, 55.1% are undergoing psychotherapeutic treatment, 44.6% have a diagnosed anxiety disorder, and 26.7% have been diagnosed with depression. Moreover, 22.5% are using psychotropic medication, and 18.7% have taken a leave of absence from work due to emotional distress—all characteristics are shown in Table 1.

**Table 1:** Sociodemographic characteristics of the sample (*n* = 610).

**Variables**		***n* (%)**
Sex	Female	531 (87.0)
	Male	79 (13.0)
Marital status	Single	271 (44.4)
	Married	339 (55.6)
Region in which you live^a^	Northeast	204 (33.4)
	North	45 (7.4)
	Southeast	151 (24.8)
	South	147 (24.1)
	Midwest	63 (10.3)
Income	Low	337 (55.2)
	Middle	179 (29.3)
	High	94 (15.4)
Have more than one job	No	268 (43.9)
	Yes	342 (56.1)
Training	Graduate	84 (13.8)
	Specialization	420 (68.9)
	MSc	84 (13.8)
	PhD	22 (3.6)
Work activity	Employed	187 (30.7)
	Self-employed worker	285 (46.7)
	Both	138 (22.6)
How satisfied are you professionally	Very much	112 (18.4)
	Quite	323 (53.0)
	Indifferent	32 (5.2)
	Little	121 (19.8)
	Very little	22 (3.6)
Would you change your profession	Yes	146 (23.9)
	No	464 (76.1)
Psychotherapy	Yes	336 (55.1)
	No	274 (44.9)
Psychotropic medication use	Yes	137 (22.5)
	No	473 (77.5)
Anxiety diagnosis	Yes	272 (44.6)
	No	338 (55,4)
Depression diagnosis	Yes	163 (26.7)
	No	447 (73.3)
Temporary removal from work for emotional distress	Yes	114 (18.7)
	No	496 (81.3)
Working hours per week, mean (SD)		31.9 (13.8)
Time working in the area in years, mean (SD)		12.2 (9.4)

^a^Brazil is divided into five major regions.

### 3.2 Exploratory factor analysis

The semantic validation of the WRQoL scale resulted in a 24-item version, which was approved by the focus group and subsequently subjected to factor analysis. The Kaiser-Meyer-Olkin (KMO) measure was 0.91, indicating the factorability of the items, which was further supported by Bartlett's test (χ^2^ = 3317.239, Degrees of Freedom = 276, *p* < 0.001). All anti-image matrix values for each item were above 0.68, indicating good adherence to the initial model. Parallel analysis was performed, suggesting the retention of three factors (Supplementary Figure 1). The correlation between the factors was confirmed, and Oblimin rotation was applied. The exploratory factor analysis (EFA) revealed a three-factor solution, with all items having a minimum loading of 0.30 on at least one factor.

The reliability for the model tested in the EFA was checked by: Omega for WRQoL was ω = 0.94, for Factor 1 (Work related wellbeing, items: 1, 4, −7, −9, 10, 15, 17, 18, −19, 20, 21, 24) was ω = 0.94, for Factor 2 (Job/career satisfaction, items: 2, 5, 6, 14) was ω = 0.84, for Factor 3 (Work Environment, items: 3, 8, 11, 12, 13, 16, 22, 23) was ω = 0.85. Interclass Composite Coefficient of the WRQoL was 0.96, for Factor 1 was 0.92, for Factor 2 was 0.79, for Factor 3 was 0.88. Cronbach's alpha for the 24 items (α = 0.89; 95% CI: 0.88–0.91), for Factor 1 (α = 0.91; 95% CI: 0.89–0.92), for Factor 2 (α = 0.79; 95% CI: 0.75–0.83), and for Factor 3 (α = 0.81; 95% CI: 0.78–0.84).

### 3.3 Confirmatory factor analysis

CFA was conducted with Subsample 2. The model (1) proposed by the EFA was tested, with initial parameters as follows: χ^2^ = 961.517, df = 249 (*p* < 0.001), RMSEA = 0.093 (90% CI = 0.071–0.100), CFI = 0.980, TLI = 0.978, and χ^2^/df = 3.861. Item 23 had a loading below 0.30 and was therefore excluded. Model (2) was then tested, with the following initial parameters: χ^2^ = 870.889, df = 227 (*p* < 0.001), RMSEA = 0.093 (90% CI = 0.086–0.099), CFI = 0.982, TLI = 0.980, and χ^2^/df = 3.835.

Consequently, model (2) required adjustments to optimize RMSEA according to the pre-established parameters in the Methods section. The modification indices were examined, indicating error covariances in Factor 1 (items 7–19). The adjusted model parameters were as follows: χ^2^ = 646.225, df = 226 (*p* < 0.001), RMSEA = 0.075 (90% CI = 0.068–0.082), CFI = 0.988, TLI = 0.987, and χ^2^/df = 2.859.

**Figure 1:**
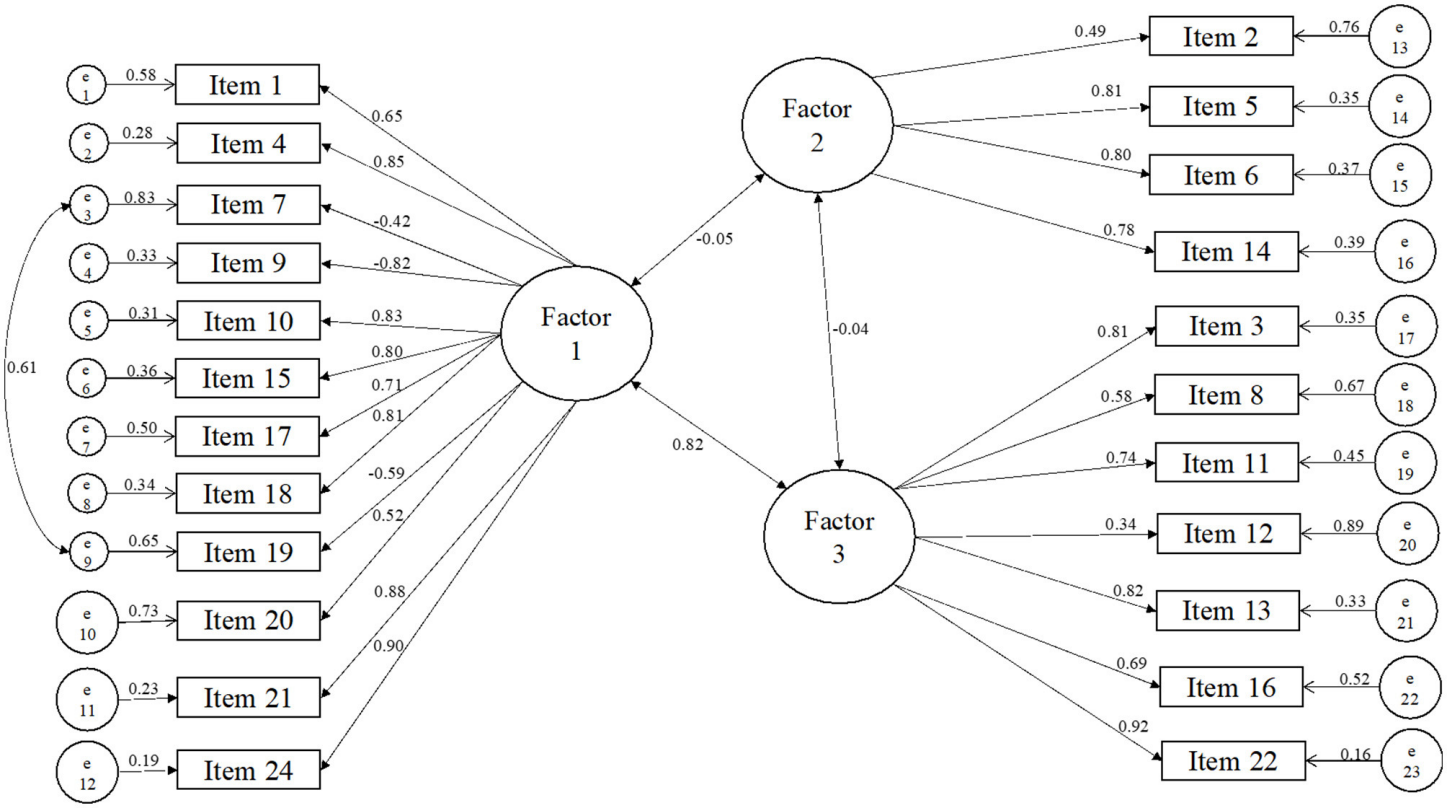
WRQoL—final model.

Additionally, we tested the original six-factor model (Van Laar et al., 2007). Despite adjustments made based on the modification indices, it did not reach the parameters outlined in the Methods section (χ^2^ = 2218.503, df = 215, *p* < 0.001; RMSEA = 0.168-−90% CI = 0.162–0.175; CFI = 0.929, TLI = 0.917, and χ^2^/df = 10.319). An ANOVA test was conducted to determine the best-fitting model between the original model and Model 2, confirming that Model 2 was a better fit for our data (Chi-Square difference = 448.48, favoring the choice of three-factor model). Furthermore, Factor 2 showed no correlation with Factors 1 and 3, which were correlated with each other (Figure 1).

### 3.4 Final reliability

We calculated the reliability using the final format confirmed by the confirmatory factor analysis and the entire sample (*n* = 610). The alpha values for the overall WRQoL scale with three factors were as follows: α = 0.89 (95% CI [0.88–0.91]) for Factor 1, α = 0.91 (95% CI [0.90–0.92]) for Factor 2, α = 0.79 (95% CI [0.76–0.81]) for Factor 3, and α = 0.82 (95% CI [0.80–0.84]) for the total scale. The Omega values for WRQoL with three factors were as follows: ω = 0.94 for the overall scale, ω = 0.93 for Factor 1 (items: 1, 4, −7, −9, 10, 15, 17, 18, −19, 20, 21, 24), ω = 0.83 for Factor 2 (items: 2, 5, 6, 14), and ω = 0.86 for Factor 3 (items: 3, 8, 11, 12, 13, 16, 22).

### 3.5 Results of the MBI, EVS, and WRQoL scales and criterion validity

The mean Life Satisfaction score (EVS) was 24.5 (±6.1) points, and 20% of the psychologists reported being dissatisfied with their lives. Additionally, 26% of the professionals experienced high levels of Emotional Exhaustion and 7.2% had high levels of Depersonalization. On the other hand, 43% of the psychologists reported high levels of Professional Accomplishment. The mean WRQoL score was 3.5 (±0.5) points. The factors on the WRQoL scale generally indicated adequate levels of work-related quality of life (see Table 2).

**Table 2:** Results of the Maslach burnout inventory (MBI), life satisfaction scale (EVS), and work-related quality of life (WRQoL) scale.

**Scales^a^**	***n* (%)**	**Median (IQR)**	**Mean (SD)**
Overall life satisfaction (5–35 points)		26.0 (8.0)	24.5 (6.1)
Dissatisfied	121 (20.0%)		
Neutral	27 (4.4%)		
Satisfied	462 (76.0%)		
MBI emotional exhaustion (9–45 points)		23.0 (9.0)	22.6 (6.6)
Low	124 (20%)		
Moderate	326 (53%)		
High	160 (26%)		
MBI depersonalization (5–25 points)		7.0 (4.8)	7.9 (2.7)
Low	244 (40%)		
Moderate	322 (53%)		
High	44 (7.2%)		
MBI professional accomplishment burnout (8–40 points)		132.0 (4.0)	32.0 (3.7)
Low	19 (3.1)		
Moderate	328 (54.0%)		
High	263 (43.0%)		
WRQoL factor 1: work-related wellbeing		3.7 (0.9)	3.5 (0.7)
WRQoL factor 2: job-career satisfaction		3.5 (1.0)	3.4 (0.8)
WRQoL factor 3: work environment		3.6 (1.0)	3.5 (0.7)
WRQoL total		3.5 (0.7)	3.5 (0.5)

^a^WRQoL range: 0–5 points. Confidence interval 0.95. IQR, interquartile range; SD, standard deviation.

We assessed the normality distribution for the continuous variables, finding that none of them followed a normal (univariate and multivariate) distribution. For criterion validity, we used measures of life satisfaction and burnout. The WRQoL showed significant correlations between its factors 1, “Work-Related Wellbeing,” and 3, “Work Environment” (rho = 0.620, *p* < 0.001). The “EVS Life Satisfaction” was significantly correlated with “Work-Related Wellbeing” (rho = 0.740, *p* < 0.001) and with “Work Environment” (rho = 0.430, *p* < 0.001). Additionally, significant correlations emerged between “Work-Related Wellbeing” and “MBI Emotional Exhaustion” (rho = −0.660, *p* < 0.001), “MBI Depersonalization” (rho = −0.340, *p* < 0.001), and “MBI Professional Accomplishment” (rho = 0.590, *p* < 0.001). Furthermore, “Work Environment” was correlated with “MBI Emotional Exhaustion” (rho = −0.400, *p* < 0.001), “MBI Depersonalization” (rho = −0.200, *p* < 0.001), and “MBI Professional Accomplishment” (rho = 0.440, *p* < 0.001). Factor 2, “WRQoL Job/Career Satisfaction,” did not show significant correlations with any of the other variables (“EVS Life Satisfaction” rho = −0.020, *p* = 0.595; “MBI Emotional Exhaustion” rho = −0.060, *p* = 0.132; “MBI Depersonalization” rho = −0.010, *p* = 0.814, and “MBI Professional Accomplishment” rho = −0.050, *p* = 0.245).

## 4 Discussion

For this study, we translated the WRQoL Scale into Brazilian Portuguese and validated this version, which showed adequate psychometric properties and evidence that the scale is relevant for assessing Work-Related Quality of Life. The Cronbach's alpha (α = 0.89) of this version was close to the original scale (α = 0.91) (Van Laar et al., 2007) and other translations (Dai et al., 2016; Garzaro et al., 2020; Gomes et al., 2013; Shabaninejad, 2012; Shukla et al., 2017). However, differences were found between the present results and the original scale and the other translated versions. In the Brazilian Portuguese version of the WRQoL scale, the following factors were confirmed: Work related wellbeing (12 items), Job/career satisfaction (four items), and Work Environment (seven items). It should also be considered that Factor 2 does not correlate with the other factors, nor does it correlate with the criterion validation measures. This suggests the possibility of excluding this factor from the WRQoL or using it as an independent subscale, given that it meets the statistical criteria for such an application.

There are some potential explanations for the structural discrepancies between the Brazilian version of the WRQoL scale and the original version in English and other languages. First, the majority of the target population consisted of self-employed professionals or employees from various institutions. Additionally, this translation marks the first adaptation for a Latin American country, which may differ from translations in European and Asian contexts due to linguistic and cultural variations (Buonanno et al., 2017). Nevertheless, the psychometric properties and structure of the Brazilian Portuguese version are well-supported by the statistical analyses conducted.

To discuss the lack of correlations between the Job/Career Satisfaction factor and the other factors and measures used in this study, it is necessary to examine the items comprising this factor. This analysis reveals that aspects such as the ability to express one's opinions, work-family balance, satisfaction with work hours, and flexibility promoted by the supervisor reflect a relationship with the work institution and the relationship the employee has with it. However, in our specific sample of psychologists, who are mainly self-employed or, when employed in institutions, do not hold this role as their primary position, such a relationship tends to be less relevant, as they have greater autonomy in managing their work. Therefore, it could be hypothesized that this factor may have a lesser or even nonexistent impact on the relationships with WRQoL, overall life satisfaction, and burnout (Hundley, 2001; Pew Research Center, 2023).

Analysis of the responses in the EVS revealed that 76% of the sample expressed satisfaction with their lives. This result likely indicates a sense of self-realization both personally and professionally, which was also observed by Moreno-Milan et al. (2019).

A significant portion of the participating professionals exhibited moderate to high levels of emotional exhaustion (EE) and depersonalization (PD), with prevalence rates of 79% and 60.2%, respectively, indicating substantial levels of burnout. These levels surpass those reported by previously research (Simionato and Simpson, 2018; Simpson et al., 2019). Emotional exhaustion (EE) emerged as the most prevalent dimension in the sample, aligning with findings in healthcare professionals such as physicians and nurses (Moss et al., 2016; Moukarzel et al., 2019; Wang et al., 2019). Despite high levels of EE and PD, a notable proportion of participants demonstrated high personal accomplishment (PA), a trend consistent with previous studies involving health professionals (Bragard et al., 2015; Werdecker and Esch, 2021; Williams et al., 2020). This suggests that even when experiencing burnout, health professionals may maintain adequate levels of PA, potentially due to the perception of contributing meaningfully to others' development and wellbeing (Råbu et al., 2016; Simpson et al., 2019).

Two of three WRQoL factors (Work-related wellbeing and Work Environment) correlated with the Burnout scale, with the negative aspects measured by the MBI showing a negative correlation with work-related quality of life. This indicates a negative association between psychologists' burnout and quality of work life, which was also found by Wang et al. (2019) in their survey of nurses. The balance between personal life and work is reported in the literature as predictive of burnout (Kotera et al., 2021; Simionato and Simpson, 2018). In other words, higher levels of burnout are associated with lower levels of work-related quality of life, indicating a moderate association between these two factors (Cetrano et al., 2017).

In literature, the negative association between WRQoL and burnout is portrayed through ergonomic problems, work pressure, and factors in the work context, such as high psychological, emotional, cognitive, and psychic demands and a lack of favorable working conditions (Cetrano et al., 2017; Cortez et al., 2019). Hence, conducting an in-depth study on the actual working conditions of psychologists could facilitate interventions aimed at enhancing their working conditions.

The “personal accomplishment” dimension of burnout and its positive correlation with the “Work-Related Wellbeing” and “Work Environment” domains of the WRQoL scale may reflect an enhanced sense of competence in fulfilling work responsibilities (Rodriguez and Carlotto, 2017). Hammond et al. (2017) highlighted the intensive work demands placed on psychologists, which necessitate additional effort and skills to manage effectively.

Consistent with previous studies (Van Laar et al., 2007; Vidal-Blanco et al., 2019), it was found that overall life satisfaction influences work-related quality of life (WRQoL). Life satisfaction is positively associated with a stronger sense of being prepared to acquire new skills (Bartosiewicz et al., 2020). In general, participants reported adequate levels of life satisfaction, despite the data being collected during the COVID-19 pandemic. This finding may be attributed to the nature of the psychological profession, as psychologists may have felt a sense of purpose and utility (Råbu et al., 2016), which was likely reinforced by the perception of contributing to the global increase in mental health needs during the pandemic (Montaño and Tovar, 2022).

Finally, this study has both limitations and strengths. A key limitation is the timing of data collection, which occurred at the onset of the COVID-19 pandemic. Several studies have highlighted changes in the mental health of healthcare professionals during this period (Rosen et al., 2020; Serrão et al., 2022). On the other hand, the study's strengths enhance the reliability of its findings. These include the diversity of activities among psychology professionals, the broad representation of professionals from across Brazil, the use of distinct sub-samples for exploratory and confirmatory factor analyses, and the application of various measure grounded in international scientific literature to assess aspects of psychologists' mental health and the criterion validity.

## 5 Conclusion

Measuring work-related quality of life presents challenges owing to the lack of a single concept and a lack of consensus among scholars regarding the factors involved. The WRQoL Scale has proven to be a good measuring instrument in several countries. The Brazilian version demonstrated adequate psychometric properties, as evidenced by its construct validity and internal consistency. This provides evidence that the WRQoL scale can be effectively used for assessing work related quality of life.

Because it is a scale based on a multidimensional concept, the instrument can be used in various work contexts. The easy access to the instrument by researchers makes it a viable option for use in research. This study was conducted with psychology professionals, and it is important to expand this research to include other occupations in the future.

In addition to validating the WRQoL scale, it is important to examine the moderate to high levels of burnout in the sample. The high levels of EE may be associated with the nature of the profession, which generally deals with people who are suffering. There is room for reflection on educational programs in colleges and universities to promote a less technical approach, including the inclusion of additional modules on self-care, social skills training, and problem-solving. It is also necessary to provide ongoing training throughout one's career, emphasizing the importance of professional organizations as support for professionals who are experiencing burnout.

## Data Availability

The dataset and analysis script are available in the repository: https://osf.io/y8v92/.
