# Supplementary material for: Burnout, life satisfaction, and work-related quality of life among psychologists
Source: Front Psychol. 2025 Feb 26;16:1532333. doi: 10.3389/fpsyg.2025.1532333 (PMC11897253; doi:10.3389/fpsyg.2025.1532333)
Supplement: Supplementary file 1 [file Supplementary_file_1.docx]

Supplementary Material

# Supplementary Figures and Tables

## Supplementary Figures

**Supplementary figure 1** – Parallel Analysis


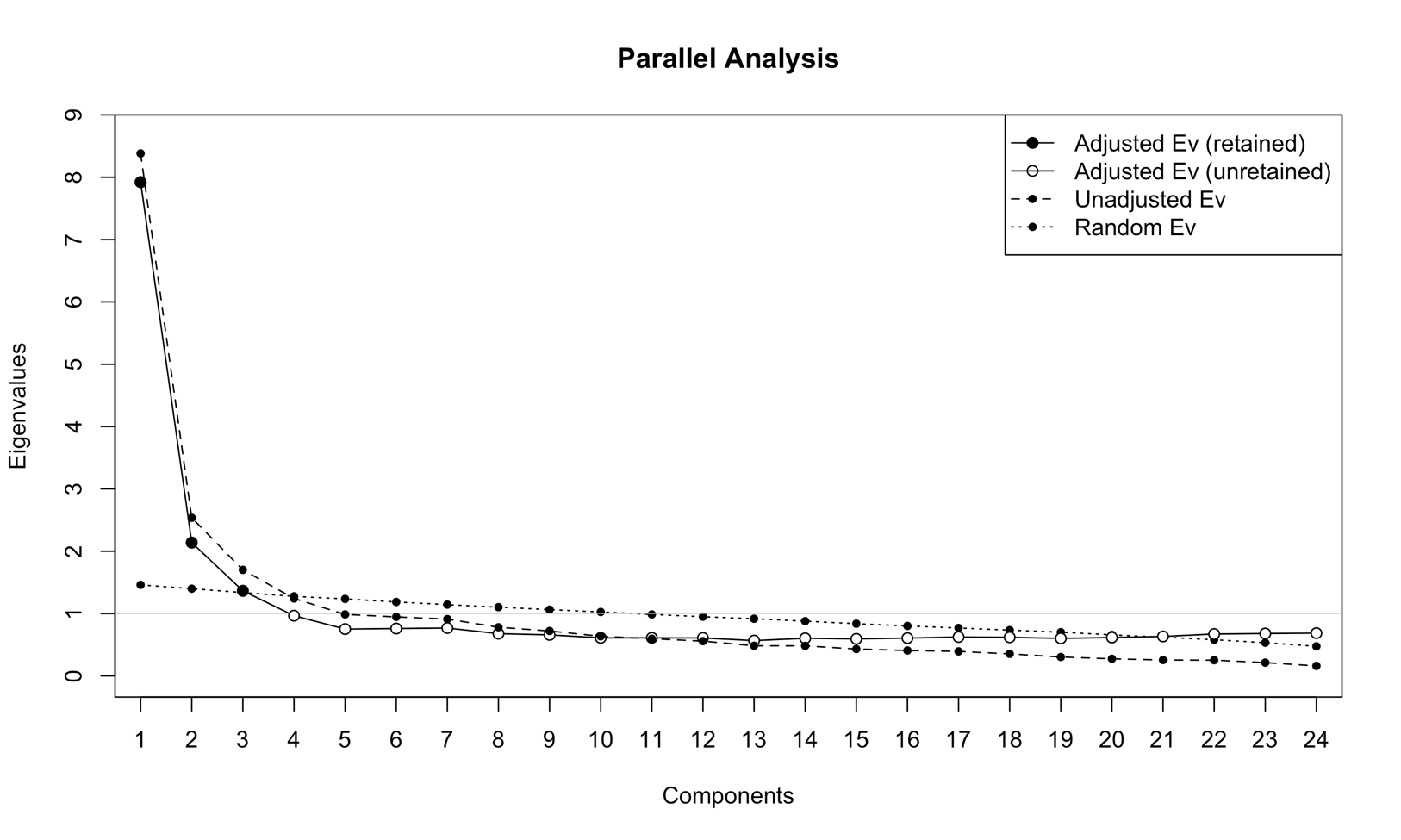


## Supplementary Tables

**Supplementary table 1** - Comparison with translations of the WRQoL scale in other languages

| Author | Country | Scale used | Sample  Calculation | Study population | Participants | Sex | Mean Age | CFA | Average overall score | Internal  Consistency |
| --- | --- | --- | --- | --- | --- | --- | --- | --- | --- | --- |
| Van Laar et al. (2007) | United Kingdom | 24 | Yes | Employees of the UK National Health Service | 953 | 86% F  14% M | ± 45 years | yes |  | α = 0.91 |
| Dai et al. (2016) | Thailand | 24 | No | Nurses | 30 in phase 1 and 213 in phase 2 | 97.7% F  2.3% M | 34.57 years | no | 4.3 (0.42); used 0–5 scale | *α* = 0.88 |
| Shabaninejad et al. (2012) | Iran | 24 | No | Family Doctors | 84 |  |  | no | 46 (12); used a scale of 0–100 | *α* = 0.78 |
| Gomes et al. (2011) | Portugal | 24 | No | Higher education professors of nursing | 183 |  |  | no |  |  |
| Shukla et al. (2017) | India | 24 | Yes | Employees of a hospital | 132 | 50.75% F 49.24% M | 31 (±8 years) | no | 4; used 0–5 scale | *α* = 0.82 |
| The present study | Brazil | 24 | Yes | Psychologists | 610 | 87% F  13% M |  | yes | 55.0 (14.32); used a scale of 0-84 | *α* = 0.94 |

Note: F = Female, M = Male;

**Supplementary table 2.** WRQoL Brazilian Portuguese – final version

| Até que ponto, você concorda com o seguinte?  Por favor, marque o espaço apropriado. | | Descordo Fortemente | Descordo | Neutro | Concordo | Concordo Fortemente |
| --- | --- | --- | --- | --- | --- | --- |
| 1 | Possuo um conjunto claro de metas e objetivos que me permite realizar meu trabalho. |  |  |  |  |  |
| 2 | Sinto que sou capaz de expressar opiniões e influenciar mudanças em minha área de trabalho. |  |  |  |  |  |
| 3 | Tenho oportunidades de utilizar minhas habilidades no trabalho. |  |  |  |  |  |
| 4 | Sinto-me bem agora. |  |  |  |  |  |
| 5 | A instituição fornece instalações adequadas e flexibilidade para que eu concilie o trabalho com a vida familiar. |  |  |  |  |  |
| 6 | Minha atual carga de trabalho e minhas condições de trabalho satisfazem minhas circunstâncias pessoais. |  |  |  |  |  |
| 7 | Frequentemente, me sinto sob pressão no trabalho. |  |  |  |  |  |
| 8 | Quando faço um bom trabalho, sou elogiado pelo meu superior. |  |  |  |  |  |
| 9 | Ultimamente, tenho me sentido infeliz e deprimido. |  |  |  |  |  |
| 10 | Estou satisfeito com minha vida. |  |  |  |  |  |
| 11 | Sou encorajado a desenvolver novas habilidades. |  |  |  |  |  |
| 12 | Em minha área de trabalho estou envolvido em decisões que me afetam. |  |  |  |  |  |
| 13 | A Instituição fornece o que preciso para fazer meu trabalho de forma efetiva. |  |  |  |  |  |
| 14 | Meu superior promove de forma ativa uma carga de trabalho e condições de trabalho flexíveis. |  |  |  |  |  |
| 15 | Na maioria dos dias, minha vida fica próxima ao ideal. |  |  |  |  |  |
| 16 | Trabalho em um local fisicamente seguro. |  |  |  |  |  |
| 17 | Geralmente as coisas terminam bem para mim. |  |  |  |  |  |
| 18 | Estou satisfeito com as oportunidades de carreira disponíveis para mim. |  |  |  |  |  |
| 19 | Frequentemente, sinto níveis de estresse no trabalho. |  |  |  |  |  |
| 20 | Estou satisfeito com a formação que recebi para realizar meu trabalho atual. |  |  |  |  |  |
| 21 | Recentemente, tenho me sentido até feliz, considerando tudo. |  |  |  |  |  |
| 22 | As condições de trabalho são satisfatórias. |  |  |  |  |  |
| 23 | No geral, estou satisfeito com a qualidade da minha vida profissional. |  |  |  |  |  |
